# Precarious employment and self-reported experiences of unwanted sexual attention and sexual harassment at work. An analysis of the European Working Conditions Survey

**DOI:** 10.1371/journal.pone.0233683

**Published:** 2020-05-28

**Authors:** Marvin Reuter, Morten Wahrendorf, Cristina Di Tecco, Tahira M. Probst, Antonio Chirumbolo, Stefanie Ritz-Timme, Claudio Barbaranelli, Sergio Iavicoli, Nico Dragano

**Affiliations:** 1 Institute of Medical Sociology, Centre for Health and Society, Medical Faculty, University of Duesseldorf, Duesseldorf, Germany; 2 Italian Workers’ Compensation Authority (INAIL), Department of Occupational and Environmental Medicine, Epidemiology and Hygiene, Monte Porzio Catone Rome, Rome, Italy; 3 Department of Psychology, Washington State University, Vancouver, Washington, United States of America; 4 Department of Psychology, Sapienza University of Rome, Rome, Italy; 5 Institute of Legal Medicine, University Hospital Duesseldorf, Duesseldorf, Germany; Free University of Bozen-Bolzano, ITALY

## Abstract

Unwanted sexual attention (UWSA) and sexual harassment (SH) are prevalent experiences for women in working life and often accompanied by poor health. Despite increasing numbers especially of young people working in insecure and irregular employment settings, there is little empirical evidence if such precarious arrangements are associated with UWSA or SH. To investigate this, we used a representative sample of the European working population consisting of 63,966 employees in 33 countries who participated in the European Working Conditions Survey in 2010 or 2015. Precarious employment (PE) was assessed on the basis of seven indicators and a formative index derived from them: temporary employment, contractual duration < 1 year, schedule unpredictability, involuntary part-time, low information on occupational health and safety risks (OSH), low pay (wage < 60%), and multiple job-holding. We measured self-reported experiences of workplace UWSA during the last month and SH during the last 12 months each using a single-item questionnaire. Multi-level Poisson regressions were used to estimate prevalence ratios for UWSA and SH according to PE adjusted for survey year, age, education, type of household, migration background, job tenure, weekly working hours, occupational position, working sector, company size, workplace gender ratio, and visiting customers or clients. 0.8% of men reported UWSA in the last month and 2.6% of the women. SH in the last year was reported by 0.4% of the men and 1.3% of the women. For both men and women, PE was significantly associated with elevated prevalence of UWSA and SH, in particular when reporting schedule unpredictability, multiple job-holding and low information on OSH. Our results suggest that precariously employed individuals may be more prone to experience unwanted sexual behaviour at the workplace compared with workers in non-precarious settings.

## Introduction

Experiences of sexual harassment (SH) or unwanted sexual attention (UWSA) can occur in all domains of daily life, but the workplace is one of the most important settings. In Europe, every third case of SH occurs at the workplace [1], while the life-time prevalence of workplace SH for women ranges between 24–50% [2–8]. Notably, workplace SH is not only an issue of discrimination and inequity, but also a cause of impaired health and job-related disadvantages. Past studies demonstrate that experiences of SH or UWSA are followed by increased levels of depressive symptoms [9–11], symptoms of post-traumatic stress disorder [12], higher blood pressure and poorer sleep [13], and higher rates of long-term sickness absence [14]. Moreover, workplace SH can have a negative impact on job satisfaction [15], and organisational commitment and productivity [16]. Women who experience SH or UWSA are also more likely to quit their job or to get discharged in the future [17,18].

In order to increase the effectiveness of prevention measures, it is important to identify which groups of workers are particularly vulnerable to UWSA and SH at the workplace. Despite growing numbers of employment arrangements that are shaped by increased insecurity, irregularity and economic vulnerability especially among young workers [19–21], little efforts have been made to investigate whether those precarious employment (PE) relations may be linked to elevated experiences of UWSA or SH. Since precariously employed workers are disadvantaged in terms of job security, statutory rights and protection against poverty, they may be more prone to experience and endure workplace SH and UWSA compared with workers in non-precarious settings. Thus, the purpose of the current study is to examine whether PE is associated with a higher prevalence of UWSA or SH. We use the two last waves of the European Working Conditions Survey (EWCS), a representative sample of the European working population, delivering detailed information about several aspects of working life. Based on previous research, we measured precarious employment by seven indicators, which imply a deviation from the permanent full-time working contract in terms of job security, regularity, protection and economic stability.

### Sexual harassment and unwanted sexual attention

During the last decades, there has been growing awareness of SH resulting in a range of laws, policies and prevention procedures worldwide. Today, workplace SH is an illegal work behaviour in 130 countries [22]. In the European context, the Istanbul Convention is an important instrument of prevention and defines SH as “unwanted verbal, non-verbal or physical conduct of a sexual nature with the purpose or effect of violating the dignity of a person, in particular when creating an intimidating, hostile, degrading, humiliating or offensive environment” [23].

Academic concepts of SH follow legal definitions and partly overlap with them [24,25]. The widely-used Sexual Experiences Questionnaire (SEQ) from Fitzgerald distinguishes between three categories of behaviours that can constitute SH [26,27]. The first is “gender harassment”, which includes verbal and nonverbal behaviours that transport insulting, hostile or degrading attitudes about women. Gender harassment is not aiming at sexual intercourse but rather at spreading misogynist attitudes. “Unwanted sexual attention” (UWSA) is the second category and includes forms of sexual advances regarded by the victim as offensive, unwanted and unreciprocated. Those advances can include requests for dates, letters, phone calls, touching, grabbing, and even severe forms of sexual assaults. The third category is “sexual coercion” or”Quid pro quo”, which is demanding sexual favours in return for job rewards or prospects.

Previous research suggests that primary targets of UWSA and SH are women [5,16], young workers [1,17], trainees [28], migrant workers [29,30], and unmarried persons [17]. There is a tendency towards women with higher education or supervising function being more likely to experience SH at work [1,31]. Further factors associated with SH or UWSA are working in sectors as construction, transport, accommodation, education, health and hospitality [2,6,8,32], and working in organisations that are dominated by individuals of the opposite sex [33].

### Precarious employment

The term “Precarious employment” describes employment forms that are considered as non-standard or atypical and associated with a lack of security, regularity, statutory rights and protection against poverty [34]. Since the end of the 1970s onwards, labour markets have substantially changed due to a range of determinants as globalisation, deregulation, growth of economic competition, advent of new technologies, economic downturns and financial crises [35]. Those developments have led to a decrease of the ‘standard employment relationship’ (SER) [36], which means a shift from permanent full-time work with regular wages and working hours to temporary, flexible and irregular employment arrangements [37]. For example, the share of workers having a temporary contract has increased in half of the OECD countries during the last 40 years [38]. In Europe, there is a growth of temporary work, involuntary part-time and low-wage employment [21,39], while the median job tenure decreases and a growing number of people are working in multiple jobs simultaneously [40,41].

For employees, the SER provided a range of important functions such as assurance of an adequate and stable income, skill development, access to social security systems, representation by independent trade unions, as well as protection against arbitrary dismissals, work accidents and safety risks [42]. The loss of those aspects comes along with elevated perceptions of job insecurity [19,20], but also with higher risks of impaired physical and mental health, occupational injuries and sickness absence [43–47].

Although a generally accepted definition of PE is still missing, there is growing agreement that PE is a multidimensional construct including various aspects simultaneously [34,40,48,49]. Modern approaches measure PE by the presence of atypical employment facets, that imply a deviation from the SER, as temporary work, low pay, involuntary part-time, schedule unpredictability, lack of protection against occupational health and safety risks, and multiple job-holding [50,51]. Additionally, multidimensional scales measure the simultaneous presence of PE indicators and were found to demonstrate good internal reliability and construct validity against the psychosocial work environment and perceived general and mental health [50,52–55]. We therefore decided to assess PE not only by the presence of a single indicator but also to investigate the accumulation of those indicators in the context of experiencing unwanted sexual behaviour at the workplace.

### Precarious employment—A determinant of workplace sexual harassment?

Just few studies have investigated the relationship between PE and experiences of unwanted sexual behaviour at the workplace yet. A qualitative study with 12 female migrant workers in Nepal suggested job insecurity making young women more vulnerable to workplace abuses [56]. A survey conducted with 1,101 Australian workers found unwanted sexual advances at work more common in temporary compared to permanent employment arrangements [57]. Lee, Kim and Park demonstrated in a survey with Korean employees that perceived job insecurity was significantly associated with workplace violence [58]. While these studies point to a possible link between PE and sexually connoted forms of workplace behaviour, a test of this relationship in a broader dataset including more countries and a more comprehensive approach to PE seems worthwhile.

### Aims and hypotheses

The aim of this article is to analyse whether PE is associated with UWSA and SH. We therefore test the following research hypotheses:

**H1**: PE is associated with a higher prevalence of self-reported experiences of UWSA at work
**H2**: PE is associated with a higher prevalence of self-reported experiences of SH at work

Since PE is related to lower job security, regularity, rights and protection, it could lead to individuals in those arrangements to be more prone to experience unwanted sexual behaviour and simultaneously less willing to fight against it. Taken together, we examine whether PE is associated with self-reported experiences of UWSA and SH at work in a representative sample of European employees.

## Methods

### Data

Data were used from wave 5 (2010) and wave 6 (2015) of the European Working Conditions Survey [59,60]. We decided to use the two last waves of the EWCS to avoid problems of small case numbers. The EWCS is a repeated cross-sectional study conducted by the European Foundation for the Improvement of Living and Working Conditions (Eurofound). Since 1991 the EWCS collects data on the working conditions of the population in 36 European countries. Participants of the EWCS are 15 years or older and work for pay or profit for at least one hour per week following the definition of the International Labour Organization (ILO). Both waves selected participants by drawing a multi-stage, stratified, random sample in each country. The sample size ranges from 1,000 to 4,000 (wave 5) and from 1,000 to 3,300 (wave 6) cases per country. Face-to-face interviews were carried out between January and August 2010 (wave 5) and between February and September 2015 (wave 6). The average response rate was 44% in 2010 and 43% in 2015. A five-stage process was applied for the translation and verification of the questionnaire. All questions used in the subsequent analyses were collected equally in both waves. A more detailed description of the methodology can be found in the technical reports [61,62].

### Study sample

The combined sample comprised N = 87,666 cases (2010: N = 43,816 and 2015: N = 43,850) from 36 European countries: Belgium, Bulgaria, Czech Republic, Denmark, Germany, Estonia, Greece, Spain, France, Ireland, Italy, Cyprus, Latvia, Lithuania, Luxembourg, Hungary, Malta, Kosovo, Netherlands, Austria, Poland, Portugal, Romania, Slovenia, Slovakia, Switzerland, Finland, Sweden, United Kingdom, Serbia, Croatia, Macedonia, Turkey, Norway, Albania, Montenegro. First, we excluded countries that did not participate in both years, which were Kosovo, Switzerland and Serbia, leading to N = 84,609 observations (96.5% of the initial sample). We then excluded individuals being self-employed, unemployed, retired, in full-time education or unable to work due to long-term illness or disability at the time of the survey, which led to N = 65,989 (75.3% of the initial sample). Further, we omitted employees working less than 10 hours per week and being older than 65 years, leading to a final sample of N = 63,966 (73.0%). Exclusion criteria equally affected the sample sizes of both waves (S1 Table in S1 Appendix). Although mainly women are afflicted by SH [63,64], we included both males and females, but analysed them separately.

### Variables

#### Experiences of unwanted sexual attention and sexual harassment at work

Unwanted sexual attention (“Over the last month, during the course of your work have you been subjected to unwanted sexual attention?”) and sexual harassment (“Over the past 12 months or since you started your main paid job, during the course of your work have you been subjected to sexual harassment?”) were assessed from self-reported experiences. Response options were “yes” or “no”. UWSA is a core dimension of SH and both items are highly correlated (r = .71) [65]. However, not all victims of UWSA are willing to label their experiences as SH [66–68]. By analysing both items separately, effects of self-labelling can better be precluded [69]. Further, UWSA is linked to stress experiences, regardless if it is labelled as SH or not [70,71].

#### Precarious employment

Due to the lack of a theory-based concept of precarious employment, we have defined PE using a labour market sociological approach. Here precarious employment is understood as the deviation of employment relations from the permanent, full-time working contract, the so-called ‘standard employment relationship’ (SER) [34,40,48,49]. These deviations include, for example, to work under a non-permanent working contract instead of a contract with unlimited duration. An important difference to work stress models as the demand control model is that precariousness is not defined by the content of work but just by the formal relation of between employer and employee. Relations are to be considered as precarious in case they imply a lack of employment security, regularity, protection and increased economic vulnerability. Table 1 gives an overview of the used indicators.

**Table 1:** Prevalence of precarious employment indicators in the European Working Conditions Survey in 2010 and 2015 (N = 63,966).

Dimension	Indicator		Men		Women	
			N	%	N	%
**Insecurity**	Non-permanent working contract ^1^^,^^2^^,^^3^	No	24,549	78.5	25,922	79.2
		Yes	6,707	21.5	6,788	20.8
	Contractual duration < 1 year ^4^^,^^5^	No	30,059	96.2	31,281	95.6
		Yes	1,197	3.8	1,429	4.4
**Irregularity**	Schedule unpredictability ^1^^,^^2^	No	27,239	87.1	29,415	89.9
		Yes	4,017	12.9	3,295	10.1
	Involuntary part-time ^1^^,^^5^^,^^6^	No	30,140	96.4	30,444	93.1
		Yes	1,116	3.6	2,266	6.9
**Lack of protection**	Low information on OSH ^6^^,^^7^	No	27,985	89.5	29,271	89.5
		Yes	3,271	10.5	3,439	10.5
**Economic vulnerability**	Low pay (wage < 60%) ^1^^,^^2^^,^^3^^,^^8^	No	29,589	94.7	27,534	84.2
		Yes	1,667	5.3	5,176	15.8
	Multiple job-holding ^6^^,^^9^^,^^10^	No	28,751	92.0	30,389	92.9
		Yes	2,505	8.0	2,321	7.1
**Total**			**31,256**	**100.0**	**32,710**	**100.0**

^1^Rodgers 1989,

^2^Tompa et al 2007,

^3^Olsthoorn 2014,

^4^Vives et al 2010,

^5^OECD 2019,

^6^International Labour Organization 2012,

^7^Becker & Engel 2018,

^8^Vives et al 2010,

^9^International Labour Organization 2015,

^10^Koranyi et al 2018.

Every indicator was calculated as a dichotomous variable (“yes” or “no”) to additionally determine the sum of all indicators in form of an employment precariousness score (EPS) ranging between 0 (no indicator was positive) and 7 (every indicators was positive). The EPS expresses the degree of deviation from the SER.

Non-permanent contracts were contracts of limited duration, temporary employment agency contracts or having no working contract at all against those of unlimited duration. We further distinguished employees with a contractual duration of less than 1 year from those with a higher duration or unlimited duration at all. Schedule unpredictability was positive if working schedules were completely set by the company while respondents regularly experienced changes of working schedules and were notified the same day or one day before. Involuntary part-time was given when respondents were working below 35h/week but would prefer to work more (underemployment). In many countries part-time workers have unequal access to social protection as sick pay, pension schemes and protection against dismissals [34,38]. Information on occupational safety and health risks (OSH) was low if respondents stated to feel not well informed or not at all informed about risks at work. Low information on OSH can indicate a less formalised relationship between employer and employee in terms of weakly defined working standards [72]. Low pay was given when the monthly wage or salary (originally measured on a continuous metric) was below 60% of the country-specific median. Low pay jobs are an important dimension of PE because they are dysfunctional in terms of social participation and economic protection against poverty [34,48–50]. Multiple job-holding was positive when having any other paid job next to the main paid job. There has been a debate about whether multiple job-holding is an indicator of precarious employment or not [47,73,74]. Even though multiple job-holding can be positive in terms of income and skill development, it is related to lower regularity of work, work-life balance and social insurance entitlements [75].

When forming the EPS as multidimensional score, we orientated on previous studies [50,51], but excluded indicators not representing a deviation from the SER. For example, long working hours, overtime or work on Sunday are aspects of working life that are not in conflict with a permanent full-time working contract and actually affect a wide range of employees. This also involved indicators of work stress models as control over work and decision latitude, which describe the content of work but not the formal relation between employer and employee [76,77].

#### Socio-demography and occupational factors

We considered a number of covariates including age, education, type of household, migration background, job tenure, weekly working hours, occupational position, working sector, company size, workplace gender ratio, and visits at clients or patients. Education was measured according to the 2011 International Standard Classification of Education (ISCED). Migration background was positive when the respondent or both of his or her parents were born in another country. Occupational position was determined according to the ESeC scheme (European Socio-economic Classification). The working sector was based on the 2-digit version of NACE 2.0 (*Nomenclature statistique des activités économiques dans la Communauté européenne*). Respondent’s age, job tenure and weekly working hours were measured and used as continuous variables. Working hours also include unpaid working time and overtimes. If respondents had worked for less than one year in the organisation, we coded job tenure as “0”. Since women experience SH more often in male-dominated workplaces [33], we calculated a variable that gives information about whether the workplace was dominated by workers of the same sex, opposite sex or equal.

#### Missing values

Missing values were found in 20 of the 21 variables ranging between 0.01–20.96% (S2 Table in S1 Appendix). A high proportion of missing values was found for income (20.96%) and small proportions (<3%) in the remaining variables. Of the 63,966 observations, missing information in at least one variable was found in 17,600 observations (27.51%). Little’s MCAR test provided evidence that missing data in the variables of interest were not missing completely at random (p<0.001) and that a complete case analysis (CCA) can lead to biased estimates [78]. Therefore, we decided to fill missing values using multiple imputation by chained equations (MICE) [79]. MICE imputes missing values by exploiting all available information from other variables in the data set and is the recommended procedure for missing rates higher than 5% [80]. Multiple imputation was conducted using Stata’s “mi impute chained” procedure and repeated five times with 10 iterations, respectively. As recommended, the imputation model was defined including all dependent and independent variables as well as country, survey wave and age (squared term) as auxiliary variables. A predictive mean matching (PMM) procedure was used to account for right-skewed distributional patterns (as here for income, working hours and job tenure). Estimation results were pooled. For comparison, we also performed the main analyses on the subset of n = 46,366 complete cases. We obtained similar results when the analyses were restricted to the complete cases only. Deviations between the two procedures were that in case of using MI instead of a CCA, the association between non-permanent work and SH became insignificant, and the relationship between involuntary part-time and UWSA became significant. However, directions of associations did not chance. Multiple imputation was generally more efficient as can be seen from the shorter confidence intervals (see S4 Table in S1 Appendix).

### Statistical analysis

We first described the study population in terms of socio-demographic and occupational characteristics. Then, we compared the prevalence of UWSA and SH as well as the mean EPS and by these factors. To test our hypotheses that PE is associated with a higher prevalence of UWSA and SH, we estimated a series of multi-level regression models. First, we present associations between each single indicator of PE and UWSA/SH. Second, we investigated how the accumulation of PE indicators, based on the EPS, is associated with UWSA and SH. We used a Poisson regression estimating prevalence ratios (PR) for UWSA and SH by each single indicators of PE and the EPS, respectively, adjusted for socio-demographical and occupational characteristics [81]. We used a robust variance estimation, since outcomes were dichotomous [82]. We estimated a series of multilevel regression models with random intercept for men and women separately. These models consider the hierarchical structure of the data, with individuals nested in countries and allow adjusting for country affiliation [83]. As a part of the sensitivity analyses, we tested if the link between EPS and UWSA/SH was similar in 2010 and 2015, for women and men, and for young and older workers. A Wald test was used to determine the joint significance of each interaction term [84]. Interactions between EPS and survey year, gender and age were illustrated graphically using margins plots. To test if our results were robust against different compositions of the EPS, we ran additional analyses where the EPS was composed of type of working contract, involuntary part-time and level of pay (see S1 Fig in S1 Appendix). Multi-level analysis was carried out by using the mepoisson procedure in Stata. All analyses were performed using Stata 15.1 MP (64-bit, StataCorp LLC, College Station, TX, USA).

## Results

### Sample description

The study population consisted of 31,256 men (48.9%) and 32,710 women (51.1%). The mean age was 41.5 years (SD±11.4). Respondents had an average tenure in their current job of 9.6 years (±9.6) and reported a mean number of 38.7 (±9.9) working hours per week. As shown in Table 2, women and men differed according to weekly working hours, type of household, education, occupational position and working sector.

**Table 2:** Description of the study population by socio-demographic and job-related characteristics.

Variable	Categories or range	Men		Women	
		N/Mean	%/(SD)	N/Mean	%/(SD)
**Survey wave**	2010	16,126	51.6	16,645	50.9
	2015	15,130	48.4	16,065	49.1
**Age**	15–65	41.2	(11.6)	41.8	(11.2)
**Education (ISCED)**	No/primary	1,558	5.0	1,101	3.4
	Secondary	20,632	66.0	19,522	59.7
	Tertiary	9,066	29.0	12,087	37.0
**Household**	Single, no children	4,583	14.7	4,442	13.6
	Couple, no children	10,922	34.9	11,024	33.7
	Couple with children	10,429	33.4	9,956	30.4
	Single with children	427	1.4	2,606	8.0
	Others	4,895	15.7	4,682	14.3
**Migration background**	Yes	4,162	13.3	4,323	13.2
**Job tenure (years)**	0–50	9.9	(9.8)	9.4	(9.3)
**Weekly working hours**	10–120	41.3	(9.4)	36.3	(9.9)
**Occupational position (ESeC)**	Semi- and unskilled workers	3,886	12.4	4,450	13.6
	Skilled workers	8,551	27.4	1,276	3.9
	Lower grade white-collar workers	4,462	14.3	9,032	27.6
	Higher grade blue-collar workers	1,909	6.1	1,012	3.1
	Higher grade white collar workers	2,267	7.3	3,623	11.1
	Lower salariat	6,388	20.4	9,957	30.4
	Higher salariat	3,793	12.1	3,360	10.3
**Working sector (NACE)**	Agriculture	861	2.8	394	1.2
	Industry	7,195	23.0	4,023	12.3
	Construction	3,637	11.6	425	1.3
	Transport	2,895	9.3	890	2.7
	Commerce and hospitality	5,491	17.6	7,157	21.9
	Financial services	1,105	3.5	1,371	4.2
	Other services	4,413	14.1	5,662	17.3
	Public administration	2,658	8.5	2,233	6.8
	Education	1,776	5.7	4,741	14.5
	Health	1,225	3.9	5,814	17.8
**Company size**	<10 employees	8,907	28.5	10,922	33.4
	10–249 employees	17,558	56.2	17,746	54.3
	250+ employees	4,791	15.3	4,042	12.4
**Workplace gender ratio**	Equal numbers of men and women	8,343	26.7	10,237	31.3
	Mostly same gender as respondent	20,397	65.3	19,723	60.3
	Mostly opposite gender	2,516	8.0	2,750	8.4
**Visiting customers or clients**	Yes	9,478	30.3	6,015	18.4
**Sample size**		**31,256**	**100.0**	**32,710**	**100.0**

Data source: European Working Conditions Survey (2010, 2015). N = 63,966 employees.

SD = Standard deviation,

### Prevalence of unwanted sexual behaviour and precarious employment

We describe the prevalence of UWSA and SH in Table 3. During the last month, 1.8% of the study participants reported to have experienced UWSA, whereby the prevalence was higher for women compared to men (2.6% vs. 0.8%). During the last 12 months, 0.8% reported experiences of SH, whereby the number was also higher for women (1.3% vs. 0.4%). The prevalence of both outcomes did not substantially change between 2010 and 2015. UWSA and SH did exhibit similar patterns across covariates. Both experiences were more often reported by participants who were young, highly educated, singles with or without children, respondents having a migration background, working in lower grade white-collar or higher grade blue-collar jobs and had a short job tenure. Further factors related to UWSA and SH were working in the health care or in commerce and hospitality, visiting customers or clients, and working in opposite gender-dominated workplaces. Rates of UWSA and SH by country can be found in the S1 Appendix (S3 Table in S1 Appendix).

**Table 3:** Prevalence of self-reported experiences of unwanted sexual attention (UWSA) and sexual harassment (SH) at work and means of employment precariousness score (EPS) by covariates.

	UWSA		SH		EPS	
	N	%	N	%	Mean	(SD)
**Survey wave**						
2010	563	1.7	279	0.9	0.74	(0.96)
2015	556	1.8	253	0.8	0.67	(0.94)
**Sex**						
Men	257	0.8	122	0.4	0.66	(0.90)
Women	862	2.6	410	1.3	0.76	(0.99)
**Age**						
15–29 years	368	3.1	151	1.3	1.06	(1.12)
30–44 years	483	1.9	248	1.0	0.68	(0.92)
45–59 years	251	1.0	124	0.5	0.58	(0.86)
60–65 years	17	0.5	9	0.3	0.59	(0.85)
**Education (ISCED)**						
No/primary	24	0.9	12	0.5	1.17	(1.11)
Secondary	688	1.7	325	0.8	0.77	(0.99)
Tertiary	407	1.9	195	0.9	0.54	(0.81)
**Type of household**						
Single, no children	212	2.4	114	1.3	0.72	(0.97)
Couple, no children	284	1.3	138	0.6	0.61	(0.88)
Couple with children	298	1.5	138	0.7	0.64	(0.89)
Single with children	107	3.5	54	1.8	0.84	(1.06)
Others	218	2.3	88	0.9	1.01	(1.10)
**Migration background**						
No	929	1.7	441	0.8	0.68	(0.93)
Yes	190	2.2	91	1.1	0.86	(1.06)
**Job tenure**						
< 1 year	218	2.5	89	1.0	1.49	(1.23)
1–5 years	465	2.2	226	1.1	0.80	(0.97)
> 5 years	436	1.3	217	0.6	0.45	(0.71)
**Weekly working hours**						
10–24 hours	132	2.1	66	1.1	1.57	(1.25)
25–39 hours	385	2.1	193	1.0	0.71	(0.98)
40–54 hours	532	1.5	245	0.7	0.53	(0.78)
55+ hours	70	2.0	28	0.8	0.90	(0.93)
**Occupational position (ESeC)**						
Semi- and unskilled workers	84	1.0	44	0.5	1.08	(1.14)
Skilled workers	64	0.7	25	0.3	0.71	(0.91)
Lower grade white-collar workers	441	3.3	195	1.5	0.95	(1.08)
Higher grade blue-collar workers	62	2.1	32	1.1	0.59	(0.84)
Higher grade white collar workers	80	1.4	44	0.8	0.59	(0.86)
Lower salariat	303	1.9	152	0.9	0.50	(0.78)
Higher salariat	85	1.2	40	0.6	0.42	(0.71)
**Working sector (NACE)**						
Agriculture	7	0.6	2	0.2	1.00	(1.10)
Industry	87	0.8	39	0.4	0.57	(0.83)
Construction	17	0.4	12	0.3	0.75	(0.95)
Transport	69	1.8	25	0.7	0.60	(0.86)
Commerce and hospitality	339	2.7	145	1.2	0.86	(1.04)
Financial services	43	1.7	22	0.9	0.42	(0.73)
Other services	157	1.6	84	0.8	0.85	(1.06)
Public administration	68	1.4	30	0.6	0.56	(0.86)
Education	78	1.2	36	0.6	0.66	(0.89)
Health	254	3.6	137	2.0	0.68	(0.91)
**Company size**						
<10 employees	351	1.8	183	0.9	0.94	(1.07)
10–249 employees	602	1.7	270	0.8	0.64	(0.90)
250+ employees	166	1.9	79	0.9	0.47	(0.77)
**Workplace gender ratio**						
Equal numbers of men and women	332	1.8	163	0.9	0.71	(0.98)
Mostly same gender as respondent	647	1.6	301	0.8	0.71	(0.94)
Mostly opposite gender	140	2.7	68	1.3	0.69	(0.94)
**Visiting customers or clients**						
No	805	1.7	366	0.8	0.72	(0.96)
Yes	314	2.0	166	1.1	0.67	(0.92)
**Total**	**1,119**	**1.8**	**532**	**0.8**	**0.71**	**(0.95)**

Data source: European Working Conditions Survey (2010, 2015). N = 63,966 employees. SD = Standard deviation. Continuous variables were categorised.

The EPS had a mean of 0.71 (±0.95). As shown in Table 3, we found the mean level of employment precariousness decreasing between 2010 and 2015. Furthermore, PE was associated with young age, female gender, low education, low occupational position, small company size, and short job tenure, as well as with low and high weekly working hours. Elevated PE scores were also found in jobs located in agriculture, commerce hospitality and other services. Means of EPS by country are listed in the S1 Appendix (S3 Table in S1 Appendix).

### Association between PE and unwanted sexual behaviour at work

We examined the association between PE and UWSA and SH in Table 4. In Model 1 (adjusted for survey year), we observed a higher prevalence of UWSA or SH in case of non-permanent work, low contractual duration, schedule unpredictability, low information on OSH, and multiple job-holding. However, associations with both outcomes were attenuated when controlling for socio-demographical and occupational covariates in Model 2. Among women, non-permanent employment was associated with a lower prevalence of SH. Low pay was not related to UWSA or SH. Generally, schedule unpredictability, low information on OSH and multiple job-holding were associated with UWSA and SH, even after controlling for covariates.

**Table 4:** Results of multi-level Poisson regressions for the association between precarious employment indicators and experiences of unwanted sexual attention (UWSA) and sexual harassment (SH).

	UWSA				SH			
	Men		Women		Men		Women	
	M1	M2	M1	M2	M1	M2	M1	M2
	PR (95% CI)	PR (95% CI)	PR (95% CI)	PR (95% CI)	PR (95% CI)	PR (95% CI)	PR (95% CI)	PR (95% CI)
	*p*-value	*p*-value	*p*-value	*p*-value	*p*-value	*p*-value	*p*-value	*p*-value
**Non-permanent contract** (yes vs. no)	1.65***	1.28	1.45***	1.12	1.97**	1.68*	0.95	0.71***
	(1.29–2.13)	(0.99–1.67)	(1.22–1.74)	(0.94–1.35)	(1.24–3.12)	(1.04–2.72)	(0.77–1.17)	(0.59–0.86)
	<0.001	0.061	<0.001	0.207	0.004	0.033	0.619	<0.001
**Contractual duration < 1 year** (yes vs. no)	1.91**	1.37	1.43*	1.02	1.80	1.47	1.21	0.91
	(1.18–3.08)	(0.86–2.20)	(1.05–1.94)	(0.76–1.37)	(0.63–5.13)	(0.52–4.15)	(0.82–1.77)	(0.61–1.35)
	0.008	0.185	0.023	0.872	0.273	0.462	0.331	0.638
**Schedule unpredictability** (yes vs. no)	1.96***	1.66***	2.25***	1.78***	1.81*	1.67	2.69***	2.12***
	(1.51–2.55)	(1.24–2.22)	(1.66–3.05)	(1.32–2.40)	(1.04–3.17)	(0.95–2.92)	(1.96–3.68)	(1.55–2.92)
	<0.001	<0.001	<0.001	<0.001	0.036	0.073	<0.001	<0.001
**Involuntary part-time** (yes vs. no)	1.59*	1.54	1.21	1.21	2.09	2.15	0.83	0.79
	(1.09–2.33)	(0.99–2.42)	(0.99–1.49)	(0.97–1.52)	(0.88–4.97)	(0.88–5.28)	(0.56–1.23)	(0.53–1.18)
	0.017	0.057	0.066	0.098	0.097	0.094	0.348	0.246
**Low information on OSH** (yes vs. no)	1.74**	1.61**	2.18***	2.12***	2.73***	2.57***	1.95***	1.93***
	(1.20–2.52)	(1.12–2.32)	(1.74–2.75)	(1.70–2.66)	(1.67–4.47)	(1.54–4.29)	(1.51–2.53)	(1.48–2.52)
	0.004	0.009	<0.001	<0.001	<0.001	<0.001	<0.001	<0.001
**Low pay (wage < 60%)** (yes vs. no)	1.29	1.04	0.94	0.96	1.38	1.13	0.82	0.84
	(0.77–2.16)	(0.62–1.74)	(0.78–1.15)	(0.79–1.18)	(0.65–2.92)	(0.54–2.35)	(0.61–1.12)	(0.60–1.19)
	0.333	0.891	0.567	0.708	0.406	0.749	0.213	0.328
**Multiple job-holding** (yes vs. no)	2.66***	2.40***	1.78***	1.68***	2.86***	2.53***	1.77***	1.65**
	(1.89–3.73)	(1.71–3.35)	(1.44–2.20)	(1.34–2.10)	(1.82–4.50)	(1.65–3.89)	(1.27–2.46)	(1.16–2.36)
	<0.001	<0.001	<0.001	<0.001	<0.001	<0.001	<0.001	0.005
**EPS** (0–7)	1.48***	1.39***	1.32***	1.28***	1.61***	1.58***	1.22***	1.17*
	(1.34–1.64)	(1.25–1.54)	(1.24–1.42)	(1.19–1.39)	(1.36–1.90)	(1.30–1.92)	(1.11–1.34)	(1.04–1.33)
	<0.001	<0.001	<0.001	<0.001	<0.001	<0.001	<0.001	0.011

Data source: European Working Conditions Survey (2010, 2015). N = 63,966 European employees (31,256 men and 32,710 women).

PR = Prevalence ratio (PR) and 95% confidence interval. EPS = Employment precariousness score (formative index of single indicators).

Model 1 adjusted for survey wave.

Model 2 additionally adjusted for age, education, type of household, migration background, job tenure, weekly working hours, occupational position, working sector, company size, workplace gender ratio, and visiting customers or clients.

* p<0.05,

** p<0.01,

*** p<0.001.

As indicated by the EPS term, the accumulation of indicators was associated with a higher frequency of self-reported experiences of UWSA and SH for both men and women. This relationship was attenuated when controlling for socio-demographic and occupational factors, but remained significant. Therefore, we could confirm both of the hypotheses. The more aspects of PE were simultaneously given, the higher was the prevalence of UWSA and SH. Especially for men, we found the link between EPS and both outcomes also stable against different compositions of the EPS (S1 Fig in S1 Appendix).

**Fig 1:**
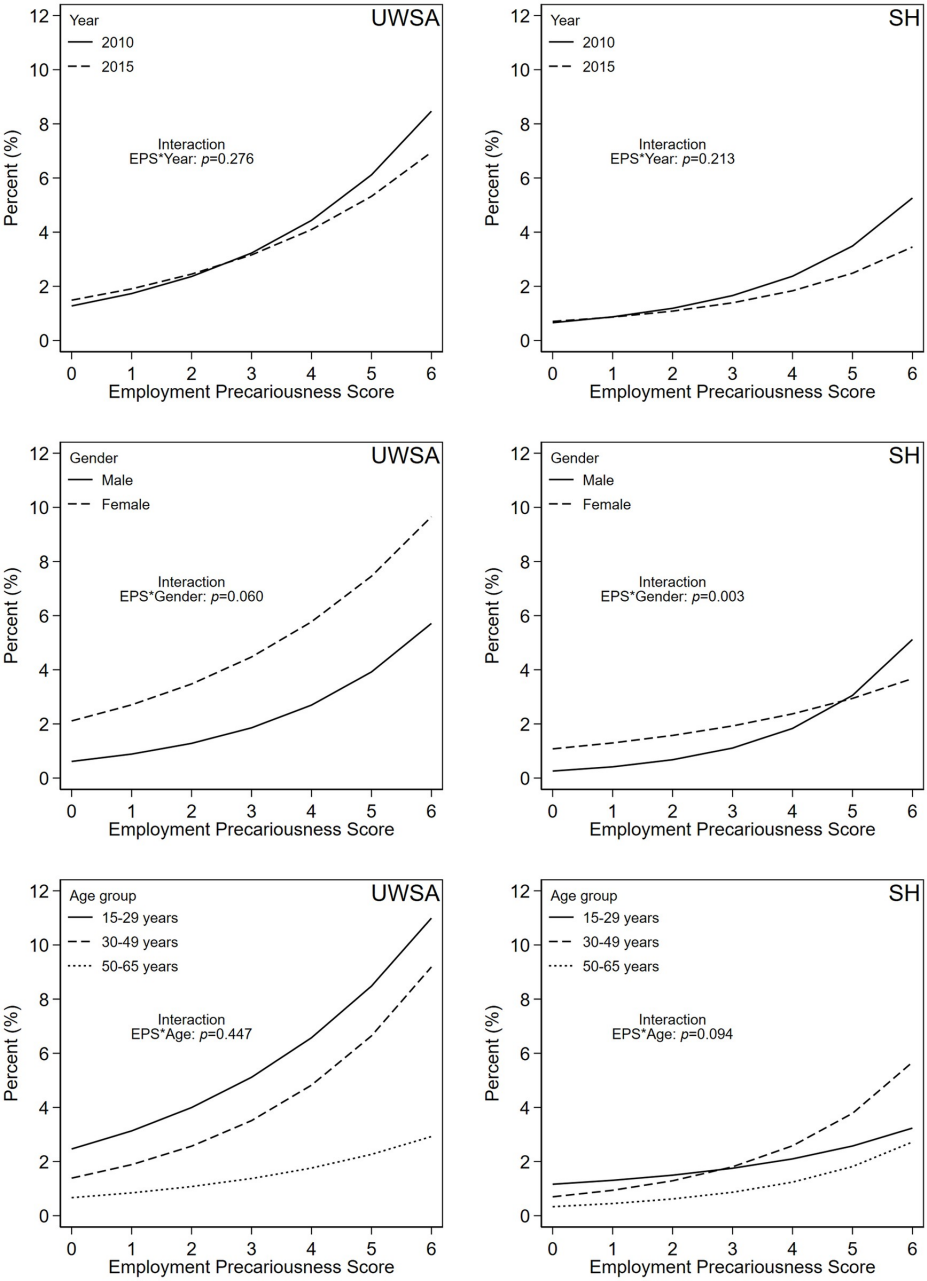
Predicted margins indicating the relationship between employment precariousness and experiences of unwanted sexual attention (UWSA) and sexual harassment (SH) at work by year, gender and age. Data source: European Working Conditions Survey (2010, 2015). N = 63,966 European employees (n = 31,256 men, n = 32,710 women). Estimates are based on multilevel regression analysis. Prevalence adjusted for survey wave, age, education, type of household, migration background, job tenure, weekly working hours, occupational position, working sector, company size, workplace gender ratio, and if the job includes visiting clients or customers. EPS range was reduced, because there were just few cases with a score of 7.

Fig 1 depicts the graphical illustration of the link between the EPS and UWSA/SH by survey year, gender and age group, respectively. We found the link between EPS and both outcomes not significantly differing between 2010 and 2015. Although women did more often report UWSA and SH, the association with precarious employment was stronger for men. However, gender differences were significant only in case of SH. There were also differences between young, middle-aged and older workers. For UWSA, the interaction between EPS and age was not significant, whereby the link seemed to be less strong in case of older workers. For SH, the association was stronger for middle-aged workers compared to young and older workers.

In summary, we found a significant association between PE and UWSA/SH for men and women. This relationship was especially robust against socio-demographical and occupational factors for schedule unpredictability, multiple job holding, low information on OSH and for the cumulative score. However, between single indicators differences were observed. For non-permanent employment, low contractual duration and involuntary part-time associations were not robust against adjustment for covariates.

## Discussion

### Main results

In this study, we investigated the relationship between PE and experiences of unwanted sexual behaviour at work. To our knowledge, no study has yet researched this potential and important variation of UWSA and SH caused by one of the most incisive labour market changes in a representative sample of the European working population. To answer our research question, we observed especially schedule unpredictability, multiple job-holding, low information on OSH, and the accumulation of PE indicators associated with a higher prevalence of self-reported UWSA and SH. Thus, our result confirms findings from past studies stemming from the Australian and Korean context [57,58].

We want to discuss three mechanisms that possibly underlie the relationship between PE and unwanted sexual behaviour. First, since PE comes along with elevated threats of job loss [19,20], precariously compared to non-precariously employed workers may be more afraid that job loss would be a consequence of complaining about SH. As a reaction to this, endurance of harassment may be an increased response. In case of women, a study found that half the employees that experience SH and filed a formal complaint were fired afterwards [18]. Another study investigating individual responses to SH found that not complaining is a common reaction either through fear or when no resources of help are available [17]. A second pathway could be that PE generally comes along with lower institutional regulation and more weakly defined working standards [72,85,86]. Workers in PE may be kept from stopping sexual harassment because they have less access to systems of formal complaint. Past research has already shown that workplace policies and mechanisms to report incidents of SH are an important tool in fighting and preventing SH [28]. In line with this, we found in particular lacking information on OSH associated with UWSA and SH. Third, a higher prevalence of UWSA and SH in PE may be due to a higher level of fluctuation of the workforce, which also leads to a higher degree of anonymity. Perpetrating UWSA or SH might be considered less risky if the victim is a non-permanent employee and less likely to complain. This may also explain the finding that multiple job-holding was associated with elevated prevalence of SH and UWSA.

Further, we found 0.8% of the men and 2.6% of the women reporting experiences of UWSA during the last month. The prevalence of SH during the last 12 months was 0.4% among men and 1.3% among women. As a result, at most half of men and women exposed to UWSA interpret their experiences as sexual harassment, which is in line with other studies investigating labelling behaviour [66–68]. Victims tend more to use the term “sexual harassment” to describe their experiences in the case of serious assaults, supportive workplace policies, or if the perpetrator has a weaker professional or social position. Additionally, frequencies of UWSA and SH can be corroborated with those reported in the 2011 Korean Working Conditions Survey (KWCS), whose methodology and questionnaire was very similar to the EWCS [58].

### Limitations

Limitations of this study result from the measurement of the outcome and the cross-sectional study character. Despite single-item questions of UWSA correlating well with behavioural categories [65], they are more likely to suffer from interpersonal differences in perception of SH. However, since workers in PE may be less likely to percept experiences of sexual attention as harassment, we could even underestimate an association in our analyses. Second, since the EWCS questionnaire does not include information on whether the perpetrator of sexual harassment was a colleague, boss or client, we cannot eliminate variations of SH caused by the fact that some occupations imply more contact with individuals (e.g. police officers, nurses, safety staff) and some imply less contact. However, we controlled to some degree for this problem by adjusting analyses for working sector, workplace gender ratio and if the job includes visits at customers or clients. Additionally, our analyses are just based on UWSA and SH without having the possibility to look at the third form of SH: Quid pro quo, which would be very interesting in context of PE. Third, since only cross-sectional data were available, it must be debated as to whether this association is also causal in nature. However, because we did not use subjective measures as perceived job insecurity in our concept of PE, we were able to control for reversed causality to some degree because indicators as working contract, income, information on OSH, predictability of working times, and multiple job-holding were less likely to be determined by experiences of UWSA or SH. Therefore, although no longitudinal survey design was used, reversed causality was attempted to be precluded to the extent possible.

### Conclusion

In conclusion, this study adds evidence to the link between precarious employment and experiences of unwanted sexual behaviour at work. Prevention measures and future research dealing with sexual harassment should consider that workers in PE relations may be a group of special risk to become a victim of UWSA and SH. Finally, the experience of UWSA and SH at work may be pathways through which precarious employment affects health.

## Supporting information

S1 Appendix (DOCX)
