# Supplementary material for: Precarious employment and self-reported experiences of unwanted sexual attention and sexual harassment at work. An analysis of the European Working Conditions Survey
Source: PLoS One. 2020 May 28;15(5):e0233683. doi: 10.1371/journal.pone.0233683 (PMC7255602; doi:10.1371/journal.pone.0233683)
Supplement: S1 Appendix — (DOCX) [file pone.0233683.s001.docx]

# Appendix

S1 Table. Drop-out criteria applied to form the study sample.

|  | **Total** | |  | **2010** | |  | **2015** | |
| --- | --- | --- | --- | --- | --- | --- | --- | --- |
|  | **N** | **%** |  | **N** | **%** |  | **N** | **%** |
| Original sample | 87,666 | 100.0 |  | 43,816 | 100.0 |  | 43,850 | 100.0 |
| Only countries participating in both years^1^ | 84,609 | 96.5 |  | 42,798 | 97.7 |  | 41,811 | 95.4 |
| Only employees, no self-employed | 68,579 | 78.2 |  | 34,572 | 78.9 |  | 34,007 | 77.6 |
| Drop unemployed, retired, education or LTI | 65,989 | 75.3 |  | 33,597 | 76.7 |  | 32,392 | 73.9 |
| Working a minimum of 10 hours/week | 64,727 | 73.8 |  | 33,142 | 75.6 |  | 31,585 | 72.0 |
| Age between 15-65 years | 63,966 | 73.0 |  | 32,771 | 74.8 |  | 31,195 | 71.1 |
| **Final sample** | **63,966** | **100.0** |  | **32,771** | **51.2** |  | **31,195** | **48.8** |

Data source: European Working Conditions Survey (2010, 2015). LTI=Long-term illness or disability. ^1^Kosovo, Switzerland and Serbia.

S2 Table. Frequency of missing values among dependent and independent variables.

| **No** | **Variable** | **Name** | **Missing (N)** | **Total (N)** | **Missing (%)** |
| --- | --- | --- | --- | --- | --- |
| (1) | female | Female | 6 | 63,966 | 0.01 |
| (2) | age | Age | 0 | 63,966 | 0.00 |
| (3) | edu | Education | 182 | 63,966 | 0.28 |
| (4) | jobten | Job tenure | 722 | 63,966 | 1.13 |
| (5) | wrkhours | Weekly working hours | 1,064 | 63,966 | 1.66 |
| (6) | nace | Working sector (NACE) | 496 | 63,966 | 0.78 |
| (7) | esec | Occupational position (ESeC) | 244 | 63,966 | 0.38 |
| (8) | estm | Company size | 1,640 | 63,966 | 2.56 |
| (9) | househ | Type of household | 126 | 63,966 | 0.20 |
| (10) | jobinc | Income (% of country median) | 13,406 | 63,966 | 20.96 |
| (11) | osh | Information on occupational safety and health | 908 | 63,966 | 1.42 |
| (12) | unpredict | Schedule unpredictability | 277 | 63,966 | 0.43 |
| (13) | parttime | Involuntary part-time | 1,064 | 63,966 | 1.66 |
| (14) | contract | Type of employment contract | 225 | 63,966 | 0.35 |
| (15) | condur | Contractual duration | 865 | 63,966 | 1.35 |
| (16) | multjob | Multiple job holding | 257 | 63,966 | 0.40 |
| (17) | visit | Job involves visiting customers or clients | 245 | 63,966 | 0.38 |
| (18) | migr | Migration background | 175 | 63,966 | 0.27 |
| (19) | sexratio | Workplace gender ratio | 325 | 63,966 | 0.51 |
| (20) | sexhar | Experiences of sexual harassment | 138 | 63,966 | 0.22 |
| (21) | sexatt | Experiences of unwanted sexual attention | 110 | 63,966 | 0.17 |

S3 Table. Mean and SD of employment precariousness score (EPS) and prevalence of unwanted sexual attention (UWSA) and sexual harassment (SH) in the EWCS by country.

|  |  |  | **EPS** | |  | **UWSA** |  | **SH** |
| --- | --- | --- | --- | --- | --- | --- | --- | --- |
| **Country** | **N** |  | **Mean** | **(SD)** |  | **%** |  | **%** |
| Belgium | 5,239 |  | 0.64 | (0.91) |  | 2.0 |  | 0.9 |
| Bulgaria | 1,699 |  | 0.51 | (0.84) |  | 0.7 |  | 0.1 |
| Czech Republic | 1,515 |  | 0.62 | (0.87) |  | 2.4 |  | 1.5 |
| Denmark | 1,667 |  | 0.62 | (0.93) |  | 2.5 |  | 1.1 |
| Germany | 3,295 |  | 0.63 | (0.91) |  | 2.2 |  | 1.2 |
| Estonia | 1,616 |  | 0.60 | (0.84) |  | 2.1 |  | 0.3 |
| Greece | 1,242 |  | 0.97 | (1.08) |  | 1.9 |  | 1.7 |
| Spain | 3,428 |  | 0.98 | (1.20) |  | 1.0 |  | 0.5 |
| France | 3,763 |  | 0.80 | (1.07) |  | 1.7 |  | 0.9 |
| Ireland | 1,544 |  | 0.89 | (1.06) |  | 2.1 |  | 1.4 |
| Italy | 1,930 |  | 0.73 | (1.07) |  | 1.3 |  | 0.3 |
| Cyprus | 1,575 |  | 0.89 | (0.88) |  | 1.3 |  | 0.4 |
| Latvia | 1,620 |  | 0.65 | (0.87) |  | 1.4 |  | 0.4 |
| Lithuania | 1,653 |  | 0.48 | (0.82) |  | 1.5 |  | 0.7 |
| Luxembourg | 1,645 |  | 0.59 | (0.85) |  | 2.8 |  | 1.6 |
| Hungary | 1,578 |  | 0.53 | (0.85) |  | 0.5 |  | 0.2 |
| Malta | 1,677 |  | 0.83 | (0.89) |  | 0.8 |  | 0.7 |
| Netherlands | 1,493 |  | 0.70 | (0.96) |  | 3.0 |  | 2.5 |
| Austria | 1,555 |  | 0.62 | (0.85) |  | 1.7 |  | 1.3 |
| Poland | 1,836 |  | 0.71 | (0.90) |  | 1.7 |  | 0.3 |
| Portugal | 1,397 |  | 0.77 | (1.01) |  | 0.4 |  | 0.5 |
| Romania | 1,552 |  | 0.50 | (0.78) |  | 1.2 |  | 0.3 |
| Slovenia | 2,368 |  | 0.54 | (0.85) |  | 1.6 |  | 0.3 |
| Slovakia | 1,598 |  | 0.57 | (0.85) |  | 1.7 |  | 0.6 |
| Finland | 1,561 |  | 0.66 | (0.95) |  | 4.4 |  | 2.0 |
| Sweden | 1,664 |  | 0.54 | (0.90) |  | 2.7 |  | 1.4 |
| United Kingdom | 2,520 |  | 0.64 | (0.88) |  | 2.3 |  | 1.1 |
| Croatia | 1,639 |  | 0.60 | (0.88) |  | 1.3 |  | 0.4 |
| FYROM | 1,391 |  | 0.86 | (0.97) |  | 0.2 |  | 0.1 |
| Turkey | 2,600 |  | 1.04 | (0.90) |  | 1.7 |  | 0.6 |
| Norway | 1,778 |  | 0.62 | (0.95) |  | 3.0 |  | 1.4 |
| Albania | 941 |  | 1.07 | (0.97) |  | 1.6 |  | 0.3 |
| Montenegro | 1,387 |  | 0.93 | (0.99) |  | 0.7 |  | 0.7 |
| **Total** | **63,966** |  | **0.71** | **(0.95)** |  | **1.8** |  | **0.8** |

Data source: European Working Conditions Survey (2010, 2015).

S4 Table. Comparing results of the multilevel regression analyses by different handling procedures for missing values.

|  |  | **Unwanted sexual attention** | |  | **Sexual harassment** | |
| --- | --- | --- | --- | --- | --- | --- |
|  |  | **CCA** | **MI** |  | **CCA** | **MI** |
|  |  | **PR  (95% CI) *p*-value** | **PR  (95% CI) *p*-value** |  | **PR  (95% CI) *p*-value** | **PR  (95% CI) *p*-value** |
|  |  |  |  |  |  |  |
| **Non-permanent contract** | | 1.10 | 1.17 |  | 0.77* | 0.90 |
|  | (yes vs. no) | (0.91-1.33) | (1.00-1.37) |  | (0.60-0.98) | (0.73-1.11) |
|  |  | 0.327 | 0.052 |  | 0.037 | 0.317 |
|  |  |  |  |  |  |  |
| **Contractual duration < 1 year** | | 1.01 | 1.11 |  | 0.95 | 1.03 |
|  | (yes vs. no) | (0.75-1.36) | (0.84-1.46) |  | (0.63-1.45) | (0.64-1.64) |
|  |  | 0.950 | 0.470 |  | 0.824 | 0.910 |
|  |  |  |  |  |  |  |
| **Schedule unpredictability** | | 1.91*** | 1.76*** |  | 2.22*** | 2.01*** |
|  | (yes vs. no) | (1.44-2.53) | (1.39-2.24) |  | (1.67-2.95) | (1.55-2.61) |
|  |  | <0.001 | <0.001 |  | <0.001 | <0.001 |
|  |  |  |  |  |  |  |
| **Involuntary part-time** | | 1.12 | 1.25* |  | 1.05 | 0.96 |
|  | (yes vs. no) | (0.87-1.43) | (1.02-1.54) |  | (0.68-1.63) | (0.62-1.48) |
|  |  | 0.388 | 0.033 |  | 0.818 | 0.845 |
|  |  |  |  |  |  |  |
| **Low information on OSH** | | 1.93*** | 1.99*** |  | 2.04*** | 2.07*** |
|  | (yes vs. no) | (1.62-2.29) | (1.68-2.36) |  | (1.59-2.61) | (1.59-2.70) |
|  |  | <0.001 | <0.001 |  | <0.001 | <0.001 |
|  |  |  |  |  |  |  |
| **Low pay (wage < 60%)** | | 0.93 | 0.95 |  | 0.88 | 0.88 |
|  | (yes vs. no) | (0.75-1.16) | (0.77-1.17) |  | (0.63-1.24) | (0.66-1.18) |
|  |  | 0.516 | 0.630 |  | 0.463 | 0.400 |
|  |  |  |  |  |  |  |
| **Multiple job-holding** | | 1.80*** | 1.84*** |  | 1.67** | 1.87*** |
|  | (yes vs. no) | (1.46-2.22) | (1.52-2.23) |  | (1.17-2.37) | (1.40-2.50) |
|  |  | <0.001 | <0.001 |  | 0.004 | <0.001 |
|  |  |  |  |  |  |  |
| **EPS** | | 1.29*** | 1.30*** |  | 1.25*** | 1.26*** |
|  | (0-7) | (1.20-1.39) | (1.22-1.39) |  | (1.12-1.40) | (1.14-1.41) |
|  |  | <0.001 | <0.001 |  | <0.001 | <0.001 |
|  |  |  |  |  |  |  |
| **Individuals** | | **46,366** | **63,966** |  | **46,366** | **63,966** |
| **Countries** | | **33** | **33** |  | **33** | **33** |

CCA=Complete case analysis. MI=Multiple imputation. PR=Prevalence ratio. CI=Confidence interval.

Results adjusted for covariates and based on Model 2 of Table 4 (men and women combined here).


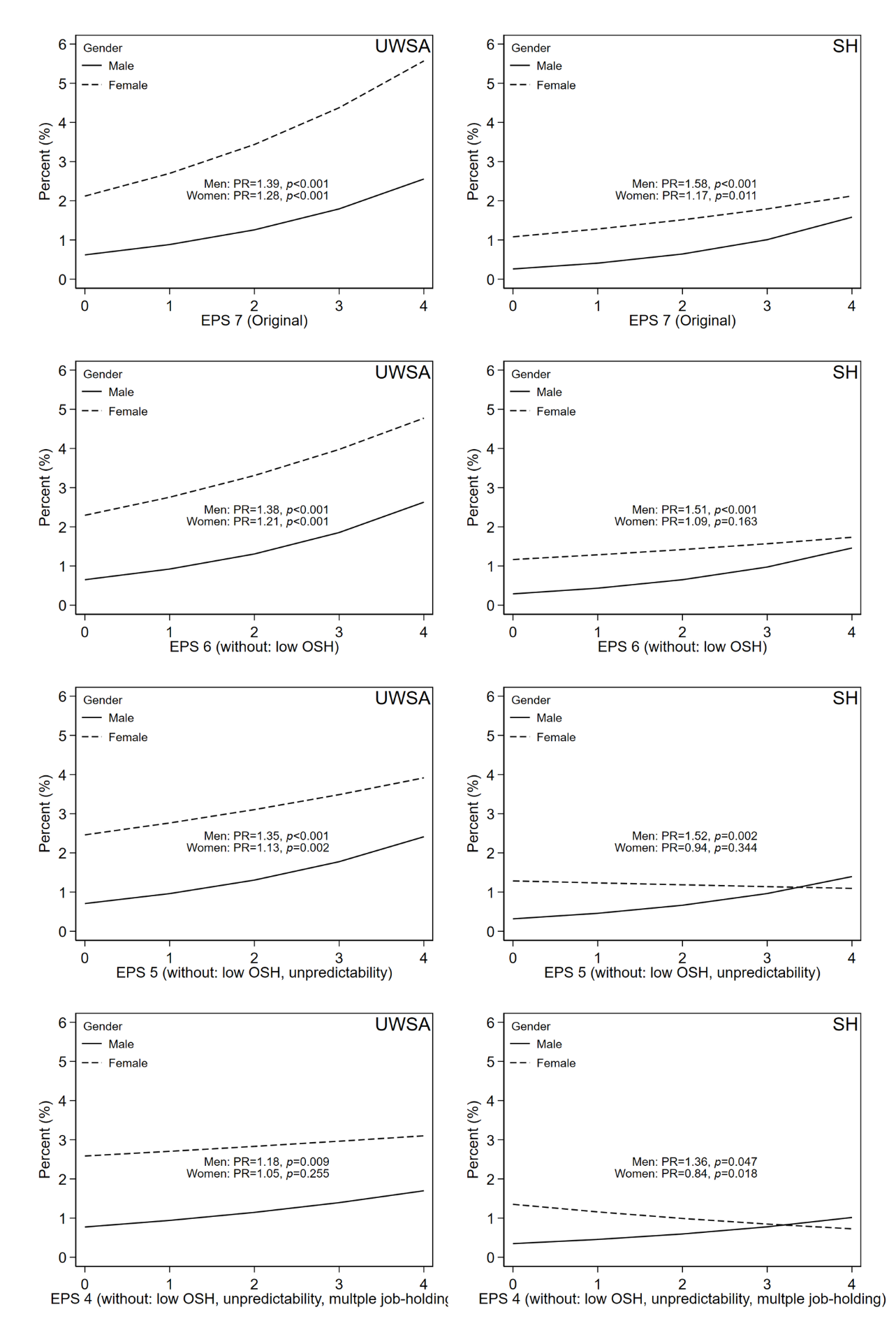


S1 Fig. Predicted margins indicating the relationship between employment precariousness and experiences of unwanted sexual attention (UWSA) and sexual harassment (SH) for different compositions of the EPS.

Data source: European Working Conditions Survey (2010, 2015). N=63,966 European employees (n=31,256 men, n=32,710 women). Estimates are based on multilevel regression analysis. Prevalence adjusted for survey wave, age, gender, education, type of household, migration background, job tenure, weekly working hours, occupational position, working sector, company size, workplace gender ratio, and if the job includes visiting clients or customers.
